# Origin, Regulation, and Fitness Effect of Chromosomal Rearrangements in the Yeast *Saccharomyces cerevisiae*

**DOI:** 10.3390/ijms22020786

**Published:** 2021-01-14

**Authors:** Xing-Xing Tang, Xue-Ping Wen, Lei Qi, Yang Sui, Ying-Xuan Zhu, Dao-Qiong Zheng

**Affiliations:** 1 Ocean College, Zhejiang University, Zhoushan 316021, China; 15700716282@163.com (X.-X.T.); 21834032@zju.edu.cn (X.-P.W.); qilei0917@zju.edu.cn (L.Q.); suiyang@zju.edu.cn (Y.S.); zyx739053039@163.com (Y.-X.Z.); 2 Department of Molecular Genetics and Microbiology, Duke University, Durham, NC 27705, USA

**Keywords:** chromosomal rearrangement, DNA repair, recombination, *S. cerevisiae*, whole-genome sequencing

## Abstract

Chromosomal rearrangements comprise unbalanced structural variations resulting in gain or loss of DNA copy numbers, as well as balanced events including translocation and inversion that are copy number neutral, both of which contribute to phenotypic evolution in organisms. The exquisite genetic assay and gene editing tools available for the model organism *Saccharomyces cerevisiae* facilitate deep exploration of the mechanisms underlying chromosomal rearrangements. We discuss here the pathways and influential factors of chromosomal rearrangements in *S. cerevisiae*. Several methods have been developed to generate on-demand chromosomal rearrangements and map the breakpoints of rearrangement events. Finally, we highlight the contributions of chromosomal rearrangements to drive phenotypic evolution in various *S. cerevisiae* strains. Given the evolutionary conservation of DNA replication and recombination in organisms, the knowledge gathered in the small genome of yeast can be extended to the genomes of higher eukaryotes.

## 1. Introduction

Chromosomal rearrangements, including deletions, duplications, translocations, inversions, and formation of extrachromosomal circular DNA (eccDNA), are ubiquitous in cancer genomes and multiple genetic diseases. It was found that the translocation between chromosome 6 and chromosome 4, leading to the fusion of proto-oncogene 1, receptor tyrosine kinase (*ROS1*) to solute carrier family 34 member 2 (*SLC34A2*), drives lung cancer development [1], and the translocation-mediated fusion of the nucleoporin 98 kDa (*NUP98*) gene and topoisomerase (DNA) IIB 180 kDa (*TOP2B*) gene acts as a pathogenic factor in acute myeloid leukemia [2]. Recent studies have reported that eccDNA with amplified oncogenes is widespread in cancers [3,4]. The enhanced expression levels of oncogenes in eccDNA-carrying cells can result from eccDNA-mediated copy number changes as well as a higher chromatin accessibility of eccDNA due to the absence of higher-order compaction [5,6,7]. In addition to being a driver of carcinogenesis, chromosomal rearrangement also underlies the phenotypic diversification and environmental adaptability in organisms [8,9,10,11]. Multiple experimental systems have been developed in model organisms to decipher the rates, qualitative spectra, genetic dependencies, and phenotypic effects of chromosomal rearrangements. We focus here on the work performed in the yeast *Saccharomyces cerevisiae*, in which the conserved DNA repair pathways and the consequences of their defect have been well explored owing to its compact genome and the powerful genetic tools established for this organism.

## 2. Origins of Chromosomal Rearrangements

The integrity of genomic DNA of cells is constantly challenged by both endogenous and exogenous agents. Of the many different classes of DNA lesions, double-stranded breaks (DSBs) are most deleterious and can be lethal to cells if left unrepaired [12]. Nonhomologous end joining (NHEJ) and homologous recombination (HR) are two evolutionally conserved pathways for DSB repair. Below we will briefly summarize how DSBs are processed and sealed, and discuss how this process results in chromosomal rearrangements in *S. cerevisiae*.

### 2.1. End Resection of DSBs and Repair Pathway Choice

In *S. cerevisiae*, the trimeric complex Mre11-Rad50-Xrs2 (MRX) recognizes DSBs and initiates end resection. It removes oligonucleotides from the 5′ end when Sae2 is recruited, resulting in a limited length of single strand DNA (ssDNA) tracts (50–100 nt) [13,14,15]. Mre11 is a member of the lambda phosphatase family, exhibiting an ssDNA endonuclease activity and a 3′ to 5′ dsDNA exonuclease activity that processes DSB ends. MRX also recruits 5′ to 3′ exonuclease Exo1 or ssDNA endonuclease Dna2 to enable extensive resection (thousands of nucleotides or even longer) in concert with the RecQ family DNA helicase Sgs1 [15,16]. What is the purpose of extensive resection? Firstly, extensive resection is critical for template searching during homology-dependent repair [13,14,16,17]. Secondly, extensive resection ensures fidelity by preventing repair between short dispersed repeats [16,17]. Thirdly, long ssDNA tails are required for cells to signal DSBs and activate DNA damage checkpoints [17]. Nevertheless, it should be noted that long 3′ ssDNA tracts facilitate multiple template invasion and complex chromosome rearrangements [18,19].

NHEJ, which does not require 3′ ssDNA overhang, takes place throughout the cell cycle. In the canonical NHEJ (c-NHEJ) pathway, the heterodimer Ku70/Ku80 recognizes and binds the broken ends to prevent resection [20,21]. The DNA ends can be ligated directly by ligase IV (Dnl4) after simple end trimming (up to 4 nt) [22]. In addition to c-NHEJ, alternative end joining (alt-NHEJ) and single-strand annealing (SSA) have been identified as backup NHEJ. Alt-NHEJ, also known as micro-homology-mediated end joining (MMEJ), is initiated by limited end resection (5 to 25 nt) [23]. The exposed 3′ ssDNA of the two broken ends are subsequently annealed, followed by single-stranded gap filling and ligation. Extensive resection resulting in long stretches (>30 nt) of 3′ ssDNA is suitable for single-strand annealing (SSA) [24]. The long 3′ ssDNA tails are first bound by replication protein A (RPA) to prevent secondary structure formation, followed by Rad52-mediated annealing. Distinct from NHEJ, HR takes place in S and G_2_ phases in the presence of sister chromatids. As mentioned above, extensive resection is a prerequisite for homolog search and alignment. In HR, the long 3′ ssDNA coated by Rad51 searches for a homologous template to invade, forming a D-Loop [25]. Detailed information about how the extent of DNA end resection and other factors that determine the choice of repair pathway (including the nature of the DSB ends, cell cycle stage, and multiple repair proteins) has been discussed in other recent reviews [26,27,28,29,30].

### 2.2. Chromosome Rearrangements Result in Poor Outcomes of DSB Repair

#### 2.2.1. NHEJ-Associated Chromosomal Rearrangements

**Figure 1:**
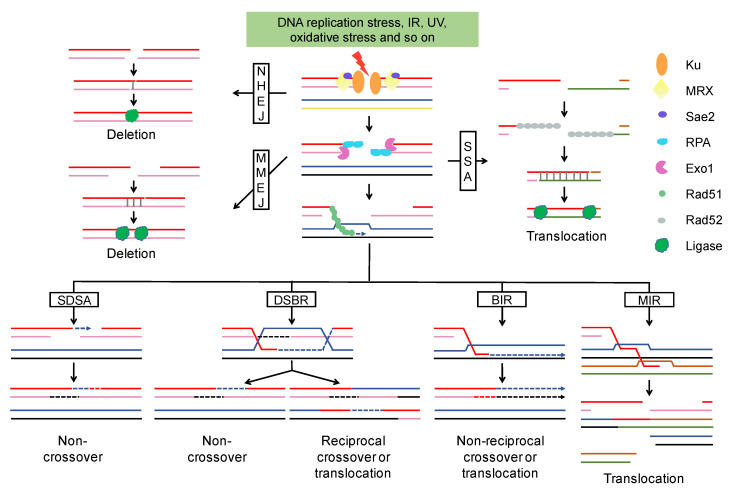
DSB end resection and repair pathway. After DSB formation, Ku binds to the DSBs and promotes the classic NHEJ pathway. NHEJ generally cause small deletions. The MRX-Sae2 complex initiates the resection and Exo1 extends the resection to expose longer 3′ ssDNA that is bound by RPA to prevent degradation. MMEJ and SSA are two alternative end-joining pathways, both of them can result in deletion or translocation. In the homologous recombination pathway, the 3′ ssDNA is coated with Rad51 and invades the homologous template to form a D-Loop. DNA synthesis is represented by an arrow and newly synthesized DNA by a broken line. Homologous recombination leads to multiple outcomes (non-crossover, crossover, gene conversion, and translocation) depending on what templates were used and in which way the D-Loop was processed. Abbreviations: NHEJ, Nonhomologous end joining; MRX, Mre11-Rad50-Xrs2 complex; RPA, replication protein A; MMEJ, microhomology-mediated end joining; SSA, single strand annealing; SDSA, synthesis-dependent strand annealing; DSBR, double-strand break repair; BIR, break-induced replication; MIR, multi-invasion-induced rearrangement.

While c-NHEJ is a rapid and efficient way to repair DSBs, it is usually regarded as an error-prone repair pathway because it rejoins the DSB ends without the use of an intact template (Figure 1). C-NHEJ often leads to small deletions and insertions that can be easily verified and selected by frameshift reversion assays in *S. cerevisiae* [31]. C-NHEJ also readily causes large deletions and translocation [32], respectively, when two DSBs occur simultaneously at the same and different chromosomes [33,34]. Cells in which c-NHEJ is blocked are still able to repair DSBs through alt-NHEJ, which is a more error-prone pathway compared to c-NHEJ. As shown in Figure 1, alt-NHEJ inevitably results in the deletion of inter-microhomology sequences and one copy of microhomology. Interestingly, polymerase θ associated insertions were occasionally observed at the DSB sites sealed by alt-NHEJ [35,36]. Chromosomal translocations via alt-NHEJ also elevate mutagenesis of the flanking of breakpoint junctions, suggesting that alt-NHEJ could destabilize genomes by triggering chromosomal arrangements and increasing the mutation rate [37]. In contrast to alt-NHEJ, SSA requires more extensive resection that results in large deletions (Figure 1) [38]. Long repeat sequences, such as Ty retrotransposon-elements, were proposed as important mediators of SSA that caused translocation in yeast [39,40]. Ramakrishnan et al. [41] reported that inverted DNA repeats placed near a DSB can generate a dicentric chromosome or a fold-back (hairpin) structure through SSA, indicating the orientation of repeats would influence the choice and outcome of DSB repair [42].

#### 2.2.2. HR-Associated Chromosomal Rearrangements

HR starts with D-Loop formation and then proceeds via one of the following sub-pathways (Figure 1): (I) Synthesis-dependent strand annealing (SDSA). The 3′ end of the invading strand is used as a primer to extend the D-Loop through DNA synthesis. The newly copied strand is re-annealed to the other broken end to allow gap filling by a second round of DNA synthesis. (II) Double-strand break repair (DSBR). The D-Loop is stabilized via “capturing” of the second broken DSB end. The resulting double Holliday junction (dHJ) can be resolved by topoisomerase-mediated dissolution or by cleavage of the Holliday junctions. (III) Break-induced replication (BIR). The invasion results in the initiation of DNA synthesis that proceeds via a migrating bubble with asynchronous DNA synthesis distal to the point of invasion. (IV) Multi-invasion-induced rearrangement (MIR). The 3′ end invades two intact donors and stimulates translocation between the two donors without any homology.

HR, during which the broken chromosome is repaired using an intact sister chromatid or homolog as a template, is much more important than NHEJ in diploid cells of *S. cerevisiae* [43]. HR between homologous chromosomes leads to loss of heterozygosity (LOH), a common genomic alteration in cancers and diploid *S. cerevisiae* strains. SDSA and DSBR are the main pathways responsible for interstitial and terminal LOH, respectively [44]. DSBR would lead to reciprocal translocation if non-allelic homology was used as the template for DSBs repair [45]. While BIR plays a crucial role in replication fork restart and telomere maintenance due to its capability to repair one-end breaks, this pathway results in terminal LOH and translocation as well [46,47]. Additionally, half-crossovers (HC) are generated when BIR is interrupted and the migrating bubble is resolved, resulting in the fusion of donor and recipient chromosomes [47]. The remaining DSB end can invade another donor to initiate a new HC or other chromosomal rearrangements [47,48]. MIR is a newly characterized pathway that drives complex rearrangements [19]. As shown in Figure 1, MIR not only stimulates translocation, but also generates new DSBs that give rise to further chromosomal rearrangements [19,45,49,50].

In summary, although NHEJ and HR are critical for maintaining the integrity of genomic DNA, aberrant DSBs repair performed by these two pathways leads to diverse chromosomal rearrangements.

## 3. Spontaneous and Genotoxic Factor-Induced Chromosomal Rearrangements in Yeast

### 3.1. Spontaneous Chromosomal Rearrangements in the Yeast Genome

In wild-type yeast cells, the rate of chromosomal rearrangements during vegetative growth is low [43]. Most information about the rates of these events was based on the genetic assay system involving single genes or partial chromosomes [51,52]. More recently, subculturing of yeast strains over many generations for mutation accumulation (MA) followed by whole-genome sequencing has allowed a more global analysis of chromosomal rearrangements [53,54,55,56]. Sui et al. [53] performed MA experiments in a diploid *S. cerevisiae* strain (*spo11/spo11*; unable to enter meiosis), identifying chromosomal rearrangements throughout the genome by deep-coverage genome sequencing. This study detected 47 chromosomal rearrangements including 35 deletions, 12 duplications, and 1 translocation. It was calculated that spontaneous chromosomal rearrangements occur at a rate of 1.8 × 10^−4^ per cell division. This rate is one magnitude lower than that of LOH events (4.6 × 10^−3^ per cell division; gene conversion and crossover) resulted from SDSA and DSBR pathways (Figure 1) [53]. They also showed certain regions are more susceptible to rearrangements than other regions. For example, the region of 184–195 Kb (including three genes *RRN11*, *CAT2*, and *VPS71*) located between two flanking Ty elements (standing for transposons of yeast, which are dispersed repeat retrotransposons in the yeast genome) on chromosome XIII, and the region of the *RDL1/2* locus on chromosome XV are two hotspots for interstitial deletion or duplication [53]. Non-allelic recombination between two sister chromatids by the DSBR pathway might be responsible for those frequent interstitial deletions and duplications (Figure 1). SSA to repair a DSB located between two direct repeats can be an alternative mechanism underlying spontaneous interstitial deletion (Figure 1). In one subcultured isolate, they also found recombination between two directly oriented Ty transposons on chromosome III that resulted in a circular DNA molecule [53]. Most strikingly, this work demonstrated that spontaneous chromosomal rearrangements generally involve homologous recombination between non-allelic dispersed repeats in the yeast genome. In a recent study by Sampaio et al. [55], the authors showed that two LOH events coincided at two different chromosomes at rates 14- to 150-fold higher than predicted if these two events originated independently of each other. This finding suggested that multiple genomic alterations can occur simultaneously during a limited time window, possibly as short as a single cycle of cell division [55]. What is the nature of recombinogenic lesions under spontaneous conditions? The study of St. Charles and Petes indicated that most mitotic recombination events were caused by DSBs in G_1_ phase [57]. Since the frequency of recombination in *S. cerevisiae* grown under anaerobic conditions was significantly lower than that under aerobic conditions [58], oxidative stress may constitute an important factor responsible for spontaneous chromosomal rearrangements.

### 3.2. Chromosomal Rearrangements under DNA Replication Stress

The fidelity of DNA replication ensures precise genetic information passage in living organisms. At least three DNA replication polymerases (α, δ, and ε) are required to complete genome replication in all eukaryotes [59,60]. Defects in DNA polymerases, the presence of DNA lesions or secondary structure, origin re-firing, as well as limited concentrations of intracellular deoxyribonucleotide, would interrupt the normal function of replication forks and lead to DNA replication stress (DRS) that has been recognized as a hallmark in cancers [61,62,63]. Using *S. cerevisiae* models in which the levels of DNA polymerases were reduced, several studies have explored how DRS stimulates DNA lesions and chromosomal rearrangements [64,65,66,67,68]. In these studies, the expression of the genes *POL1* (encoding the catalytic subunit of polymerase α) or *POL3* (encoding the catalytic subunit of polymerase δ) was regulated by the galactose-inducible *GAL1* promoter. When grown in low-galactose medium (0.005% galactose), the expression levels of the DNA polymerases were reduced which greatly elevated rates of genomic alterations. Using the whole-genome SNP microarray, Zheng et al. [65] detected the chromosomal rearrangements among 35 colonies derived from a low polymerase δ strain. Of the 41 interstitial deletions/duplications, 27 were within the tandem clusters (*CUP1* and *HXT6/7* genes), 4 were between solo long terminal repeats (LTRs), and 10 involved nonallelic Ty transposons. They also identified repeats that mediated 16 terminal alterations and most of them were paired events: a terminal deletion and a terminal duplication deletion occurred in the same strain. This pattern may be caused by a break on one chromosome that was repaired by BIR using an ectopic allele on a nonhomologous chromosome as a template (Figure 1). This work showed that DRS imposed by low levels of polymerase δ stimulate the frequency of chromosomal rearrangement by two orders of magnitudes [65]; the main source of chromosomal rearrangements is HR between ectopic repeats rather than NHEJ under DNA replication stress. By a genetic assay, the authors also showed that most recombinogenic DNA lesions in the low polymerase δ cells occurred during S/G_2_ phase, presumably as a consequence of replication fork collapse under DNA replication stress.

### 3.3. DNA Repair Deficiency Contributes to Chromosomal Rearrangements

The DSB repair process can be broadly divided into two stages: DSB signaling and repair. These two processes coordinate to guard the genome integrity in cells [69]. In *S. cerevisiae*, Mec1-Ddc2 (orthologues of human ATR-ATRIP) and Tel1 (orthologue of human ATM) are two sensor kinases to perceive DSBs [70]. Mec1-Ddc2 senses ssDNA through interaction with RPA [71], while Tel1 is recruited to DSBs and activated by the MRX complex [72]. It was found that the *mec1 tel1* null mutant showed a great increase (~13000 fold) in chromosomal rearrangement, of which most are chromosome translocations [73,74]. Downstream gene (such as *RAD53*, *SML1*, *MRC1*, and *TOF1*) knockout increased the frequency of chromosomal rearrangement as well [75]. These studies suggest that the deficiency in DNA damage checkpoints drives chromosomal rearrangements.

Although HR is the major genetic mechanism underlying chromosomal rearrangements in *S. cerevisiae*, loss function of HR (*rad52* mutant or *rad51 rad59* double mutant) results in a higher rate of chromosomal rearrangements due to the error-prone feature of NHEJ (see review [76]). The genes involved in DSB resection, which channel DSBs into HR, are particularly important for maintaining genome stability. The deletion of either *MRE11* or *XRS2* increased the rate of chromosome rearrangement by more than 500-fold [77,78]. Large palindromic duplications occurred at a higher rate in the *sae2 mre11* double mutant than in wild-type cells [18]. The *sgs1* mutant increas the frequencies of deletions and translocations that contain extra regions of imperfect homology at the breakpoints [79]. A defect in *EXO1* caused about a 14-fold increase in chromosomal rearrangements [80], and the *exo1 sgs1* double mutant showed a dramatic increase in chromosomal rearrangement of about 450 times [81]. The HR intermediates can be disrupted by multiple pathways [82,83,84,85], among which the DNA mismatch repair pathway can unwind heteroduplex HR intermediates by recognizing mismatches by Msh2-Msh6 or Msh2-Msh3 [86,87]. Defects in the DNA mismatch repair lead to close rates of recombination mediated by identical sequences and by imperfectly matched sequences [86].

Overall, DNA repair deficiency would dramatically increase the frequency of chromosomal rearrangements. It should be noted that the overactivity of certain HR components also brings risks to genome. For example, the 3′ to 5′ exonuclease activity of Mre11 contributes to large deletions and translocations due to the excessive process of DSBs [88], emphasizing that strict regulation of the DNA repair activity is pivotal to genome stability.

### 3.4. Chromosomal Rearrangements Induced by Ion Irradiation (IR), Chemical, and Oxidative Stress

The integrity of genomic DNA is also challenged by exogenous factors that inflict damage upon DNA and induce chromosomal rearrangements [89,90,91]. Although IR is not a major force of spontaneous genomic alterations, it is extensively used as an exogenous factor to generate DNA damage and recombination in research. In the study of Argueso et al. [40], it was found that 7–28% of the G_2_-phase arrested yeast cells still survived after extensive irradiation that introduced about 250 DSBs per cell, demonstrating the ability of a eukaryotic genome to undergo extensive repair and rebuilding from extreme DSBs. Using pulsed-field gel electrophoresis (PFGE) analysis, they found nearly two-thirds of the appeared colonies (54 of 71) contained at least one chromosomal rearrangement. Comparative genomic hybridization arrays (CGHarrays) of IR-treated isolates revealed that almost all of the chromosomal rearrangements resulted from HR between nonallelic repetitive elements (mainly Ty retrotransposons). These findings argued that HR is the primary pathway to repair DSBs in diploid yeast cells resulted from IR, and after IR treatment, chromosomal rearrangements generated by recombination between nonallelic repeats can profoundly reshape the genomes [40]. Interestingly, although ultraviolet (UV) is highly recombinogenic, very few chromosomal rearrangements were observed in UV-treated yeast cells [92]. This difference is likely to reflect the different numbers of DSBs caused by IR and UV. IR is able to cleave DNA directly, causing much more DSBs than UV [92].

Bleomycin (BLM) is a radiomimetic chemical used for the treatment of a variety of tumors [93]. Freeman and Hoffmann found the frequency of mitotic recombination in yeast could be significantly elevated by BLM treatment [94]. Using a whole-genome SNP array, Sheng et al. [95] detected 78 LOH events and 3 aneuploidy events among the 13 isolates derived from a heterozygous diploid *S. cerevisiae* strain QSS4 after treatment with 4 μg/mL Zeocin (a BLM analog). The authors also detected an interstitial deletion mediated by an LTR sequence [95]. This study demonstrated that a DNA damaging chemical can greatly stimulate mitotic recombination that leads to LOH and chromosomal rearrangement in yeast [95]. The ratio of chromosomal rearrangement and the LOH event was similar to that observed in the wild type cells. Etoposide is a podophyllotoxin derivative that belongs to the class of topoisomerase poisons. Exposure to etoposide leads to ssDNA breaks by trapping the topoisomerase 2 (Top2)-DNA complex, which can be converted to DSBs after chromosomal replication [96]. It has been demonstrated that Top2 poisons can trigger chromosomal translocations and shorten the replicative lifespan of yeast [97,98]. Camptothecin (CPT) also indirectly causes DSBs by stabilizing the covalent Top1-DNA cleavage intermediate [99]. Treatment with 50 μg/mL CPT increased the frequency of chromosomal rearrangements by about 50-fold in yeast [51]. Further, copy number variations of rDNA and *CUP1* clusters were observed after CPT treatment [100]. Methyl benzimidazole-2-yl-carbamate (MBC or carbendazim) is a widely used broad-spectrum benzimidazole fungicide that interferes with the function of the mitotic spindle, resulting in whole chromosomal aberration (monosomy, trisomy, and uniparental disomy). Additionally, it was also reported that MBC treatment led to chromosomal rearrangements [101,102]. Because of its potential to stimulate large-scale genomic alterations, MBC has been used as a mutagen to obtain *S. cerevisiae* mutants with improved stress tolerance and fermentation performances in several studies [103,104]. The primary conclusion from the above discussion is that any chemical that interrupts DNA replication or separation may be a potent inducer of chromosomal rearrangements.

Oxidative stress is a constant threat to the genome stability of aerobic organisms [105]. Reactive oxidative species (ROS)-induced DNA damage includes various general damages as well as DNA breaks [106]. In the study by Zhang et al. [58], yeast cells treated with 100 mM H_2_O_2_ for 1 h resulted in a one hundred-fold elevation of the frequency of mitotic recombination in a diploid *S. cerevisiae* strain, demonstrating that H_2_O_2_ is a potent inducer of genome instability. The authors observed 10 deletions and duplications, half were interstitial and half were terminal, among 30 isolates obtained after repeated exposure to H_2_O_2_. Similarly, chromosomal rearrangement can result from mutations of genes (such as *SOD1* and *TSA2*) involved in the anti-oxidative systems [58,107] and intracellular ROS accumulation induced by extracellular stressors. Qi et al. [108] found that exposure to furfural, a major inhibitor existing in the cellulosic hydrolysate used for bioethanol fermentation, led to enhanced rates of chromosomal rearrangements in yeast cells. Although furfural cannot cleave DNA directly, the authors observed frequent in vivo DNA breaks due to accumulation of intracellular ROS [108]. It is likely that prolonged exposure of yeast cells to the recombinogenic agent under industrial conditions could quickly lead to loss of selected desirable traits of industrial yeast strains. These findings have direct implications for the use of yeast cells in the production of bioethanol from cellulosic feedstocks, particularly in distilleries that utilize cell recycling.

## 4. Methods to Generate and Map Chromosomal Rearrangements

Although chromosomal rearrangements represent common genetic polymorphisms among *S. cerevisiae* strains, the determination of the fitness impact of chromosomal rearrangements independently from the effect of DNA point mutations remains challenging. To tackle this, as described below, several technologies and analysis methods have been developed to improve the efficiency of generating and mapping targeted chromosomal rearrangements.

### 4.1. Synthetic Chromosome Rearrangement and Modification by loxP-Mediated Evolution (SCRaMbLE)

Facilitated by advances in DNA synthesis, the yeast chromosomes can be de novo resynthesized and systematically modified [109]. With symmetric loxP site (ATGTACAT) sites inserted 3 bp after the stop codon of each nonessential gene, an inducible chromosome rearrangement system defined as SCRaMbLE can be built in the synthetic yeast genome (Sc2.0; www.syntheticyeast.org). As mentioned in Table 1, this approach proved to be efficient to generate mutants with enhanced stress tolerance and metabolic capacities through massive chromosome rearrangement upon the expression of Cre recombinase that recognizes the LoxP sites [110,111,112,113,114]. For example, Luo et al. [110] illustrated the application of SCRaMbLE to yield robust mutants from a parental strain with synthesized chromosome XII. In one mutant showing higher ethanol resistance, it was identified that an inversion event that caused the function loss of the transcription factor gene *ACE2* was responsible for the improved ethanol tolerance. To aid the selection of positive SCRaMbLEd colonies, Luo et al. [110] also developed a genetic screening system based on the on/off switch of two auxotrophic markers flanked by two loxP sites. Following the induction of Cre recombinase, the marker gene cassette is inverted, resulting in one marker (*URA3*) being turned on and the other (*LEU2*) being turned off [110].

### 4.2. Generation of On-Demand Chromosomal Rearrangements

**Figure 2:**
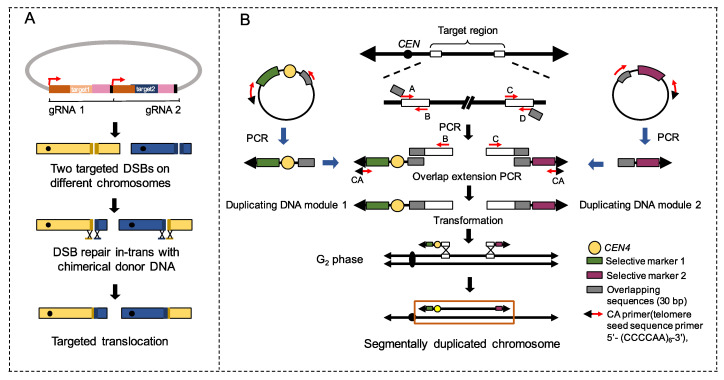
Two methods to generate on-demand chromosomal rearrangements. (**A**) Generation of targeted translocation with CRISPR-Cas9. Using two different gRNAs, two DSBs are simultaneously generated on different chromosomes by Cas9. DSBs are then repaired in trans with a chimerical donor DNA oligonucleotide, resulting in designed translocation. (**B**) PCR-mediated construction of a segmentally duplicated chromosome. The most left/right regions (400 bp; white rectangle) of a target region were amplified by PCR. The primers A, B, C, and D varied with the target site. A DNA fragment containing a centromere (*CEN4*) and the selective marker 1 cassette, paired with the PCR product that was produced by primers A and B, were used to generate “duplicate DNA module 1” by overlap extension PCR. Similarly, a DNA fragment containing the selective marker 2 and the PCR product produced by primers C and D were used to amplify “duplicate DNA module 2”. Duplicate DNA modules were then introduced into yeast cells to duplicate the selected region by homologous recombination and DNA replication.

Fleiss et al. [115] reported an application of the Clustered Regularly Interspaced Short Palindromic Repeats-CRISPR associated (CRISPR-Cas ) system to generate uniquely targeted translocation. In their protocol, two Cas9-induced DSBs were introduced on different chromosomes to force the trans-chromosomal repair through HR by chimerical donor DNAs (Figure 2A) [115]. The authors also developed a protocol to generate multiple concomitant reciprocal translocations by introducing guide RNA sequences targeted to LTRs [115]. It was demonstrated that LTR-mediated rearrangements are efficient to fuel phenotypic diversification [115]. The work of Natesuntorn et al. [116] described a PCR-mediated method to construct targeted chromosomal segments (Figure 2B). As shown in Figure 2B, two guiding “duplicating DNA modules” were first amplified by PCR and transformed into yeast cells to initiate the segmental duplication of the targeted chromosomal region by HR. Up to 300 kb of the targeted chromosomal region can be generated efficiently by this method [116]. Phenotypic assays of the segmental duplication mutants showed direct associations between segmental duplication and improved resistance against environmental stressors (see Section 5). However, since the duplicated region in this study was large (100 to 300 kb) and always contained at least dozens of genes, further work is required to verify the contribution of a specific gene or a smaller number of genes to the improved tolerance.

### 4.3. Detection of Rearrangements by Nanopore Sequencing Technology

PFGE paired with Southern blot analysis has been widely used to identify the rearrangement event in the yeast genome. The application of high-throughput analysis methods, including CGHarray [40], SNP microarray [117], and second-generation sequencing [53,54], allows mapping of chromosomal rearrangements at the whole genome level. However, second-generation sequencing lacks the ability to parse junctions if the rearrangement events are mediated by long (>300 bp) repeats due to short sequencing reads [118]. The long-read sequencing platforms have the potential to address this challenge. Researchers have reported read lengths in excess of 2 Mb using Oxford nanopore sequencing [119]. The study of McGinty et al. [118] illustrated the advantages of nanopore sequencing over other methods in identifying repeat-mediated complex chromosomal rearrangements in *S. cerevisiae*. To obtain long reads, high-molecular-weight DNA extraction and careful library preparation should be done. The “dry” part of nanopore sequencing includes base calling to obtain read sequences, mapping of reads onto the reference genome, and detection of rearrangement events. Albacore, Guppy, and Flappie are widely used for base calling of nanopore reads [120]. The combination of NGMLR (mapping reads to genome) and Sniffles (variation calling) is powerful to detect chromosomal rearrangements using nanopore reads [121]. Finally, the alignments can be visualized using Ribbon, a tool specializing in split reads [122]. It is worth mentioning that nanopore sequencing has a broad application in the detection of structure variations in human genomes as well [123]. With the improvement of base quality and mapping methods, we expect that nanopore sequencing will provide a greater opportunity for chromosomal arrangement analysis in the near future.

## 5. Phenotypic Effects of Chromosomal Rearrangements

In addition to being the best studied model organism of eukaryotes, *S. cerevisiae* is also widely used in the fields of baking, winemaking, brewing, bioethanol production, and synthetic biology. *S. cerevisiae* cells inevitably encounter stressors including high concentrations of substrates and fermentation products, metal ions, temperature fluctuation, and inhibitors (such as weak acids, furan derivatives, and phenolic compounds) during the industrial processes [124]. Stress tolerance is a key component of the yeast-based fermentation directly influencing the production efficiency [125]. Genomic studies of *S. cerevisiae* strains isolated from natural, clinical, and industrial conditions have uncovered various chromosomal rearrangement events in this species, indicating genomic structural variations are beneficial, and provide the stock for adaptive evolution under certain conditions [11,103,126,127,128]. In Table 1, we summarize the studies showing the associations of chromosomal rearrangements and phenotypic variations in *S. cerevisiae* strains.

Copper is an essential cofactor for many enzymes, but a high copper content in a medium is toxic to yeast cells during the ethanol fermentation process [129]. Chang et al. [130] found that the *S. cerevisiae* strains isolated from the Evolution Canyon, Israel, were highly resistant to copper. This phenotype mainly resulted from the recombination between chromosomes VII and VIII, which increased the copy numbers of *CUP1* and *CUP2*, two major genes involved in copper regulation [130]. Interestingly, they also observed that the chromosomal rearrangements in copper-tolerant yeast strains were highly reversible, suggesting that chromosomal rearrangement provides not only a fast arising but also readily reversible source of genomic variations during adaptive evolution [130]. Using PCR-mediated chromosome duplication technology (see Section 4.2), Natesuntorn et al. [116] constructed *S. cerevisiae* strains in which a certain chromosomal region (100–290 kb) was duplicated. Such segmental duplication was effective to improve the tolerance to high temperature (39 °C and 40 °C), lactic acid (4% and 5%), ethanol (8%), sulfuric acid (0.44%), acetic acid (80 mM), formic acid (36 mM) or NaCl (1.2 M) [116]. The authors also found the duplication of certain chromosomal segments, such as the region from 400 kb to 601 kb on chromosome II, contributed to the resistance of multiple stressors. Sugiyama et al. [131] found the deletion of the DNA region between *UBP2* and *LSC1* on chromosome XV resulted in a sensitive phenotype of hygromycin B. However, knocking out each individual ORF within the deleted region alone did not result in hygromycin B sensitivity, suggesting certain phenotypic changes can only occur when multiple genes are deleted or duplicated at the same time [131].

The DAL cluster that contains 6 DAL genes is the largest metabolic gene cluster in the yeast genome, enabling yeast to use the non-preferred nitrogen source allantoin. Naseeb and Delneri found that inversion of the *DAL2* gene (encoding allantoicase that converts allantoate to urea and ureidoglycolate) in the DAL cluster reduced the expression of *DAL2* and its neighboring genes *DAL1* (encoding allantoicase that converts allantoin to allantoate) and *DAL4* (encoding allantoin permease) as well, leading to reduced fitness in allantoin-containing medium [132]. Sulfites are a food preservative widely used in winemaking. *S. cerevisiae* can produce sulfites (H_2_S and SO_2_) during alcoholic fermentation. Resistance to sulfites is a desired trait for yeast used for winemaking [133]. Zimmer et al. [134] reported that a translocation between chromosome XV and chromosome XVI in the wine strain GN increased the expression of the sulfite pump (Ssu1p), thus resulting in a shorter lag phase time. The exact chromosomal breakpoint occurred at the position 161,342 (between *ADH1* and *PHM7*) and 373,561 (between *SSU1* and *NOG1*) for chromosome XV and chromosome XVI, respectively [134]. In the other wine strain, an inversion event mediated by the microhomology sequence between *SSU1* and *GCR1* regulatory regions led to an increase in the expression of *SSU1* and sulfite resistance [135].

SCRaMbLE has been proven powerful to enhance tolerance to high temperature, ethanol, lactic acid, and alkali in the *S. cerevisiae* strains harboring synthesized chromosomes [110,114]. This system can also be used to construct mutants with improved yields of high value-added compounds and substrate utilizing capability. Xylose is abundant in lignocellulosic biomass, while it cannot be utilized by the wild *S. cerevisiae* for growth and ethanol fermentation. Using SCRaMbLE, xylose utilization was improved in *S. cerevisiae* with a synthesized chromosome V and a plasmid harboring two genes (*XYL1* and *XYL2* from *Scheffersomyces stipitis*) involved in xylose metabolism [112]. In the same study, the authors also obtained SCRaMbLEd strains with enhanced production of violacein and penicillin G [112]. Jia et al. [111] integrated the carotenoid pathway containing gene *crtE*, *crtI*, and *crtYB,* into a haploid strain with synthesized chromosome V, resulting in a start strain yJBH000 that produced 12.53 mg/L carotenoid. The production of carotenoids was increased 1.5-fold in the yJBH000-derived mutants, in which gene *YEL013W* was lost after SCRaMbLE [111].

It is likely that certain chromosomal rearrangements can be readily selected under stressful conditions. Dunham et al. [136] reported that the evolved strains selected from glucose-limited conditions often carry an amplified region (*HXT6/7* region on chromosome IV) that encodes a high-affinity hexose transporter. Gresham et al. [137] found the amplifications of a high-affinity sulfate transporter locus (*SUL1*) can be selected, contributing to the faster growth of *S. cerevisiae* under sulfate-limited conditions. In the work of Zhang et al. [58], a H_2_O_2_-resistant mutant was selected after multiple rounds of H_2_O_2_ treatments. By whole-genome SNP microarray, the authors found chromosome VII underwent multiple in situ segmental duplications in that mutant. As a result, the mutant obtained H_2_O_2_ resistance due to the amplification of the catalase-encoding gene *CTT1* [58]. Interestingly, chronic exposure of human fibroblasts to H_2_O_2_ also resulted in the amplification of the catalase-encoding gene *CAT* and enhanced oxidative stress resistance [138]. Given the fact that ROS is inevitably produced in cellular metabolism and can be readily induced by a variety of environmental conditions [105], oxidative DNA damage may be a general factor that drives chromosomal rearrangements in eukaryotes.

How do chromosomal rearrangements fuel phenotypic alterations? Firstly, chromosomal rearrangement-associated DNA dosage changes lead to altered gene expression levels. Secondly, translocations cause gene dysregulation at translocation breakpoints and/or gene fusion [130,132]. Thirdly, rearrangements reorganize the 3D architecture of chromosomes, which in turn can modify the expression of genetic information [6,7]. Lastly, cells with large deletions/duplications, such as aneuploidy cells, may suffer from aneuploidy-associated stresses [139,140]. We refer the reader interested in the phenotypic effects of whole chromosome aneuploidy to the reviews [44,126].

**Table 1:** Studies of phenotypic effects of chromosomal rearrangements in *S. cerevisiae.*

Strains	Events	Phenotypic Effect	Reference
JSC25-1 (a diploid lab strain)	Duplication of a region that contains catalase-encoding gene *CTT1* on chrVII	Improved tolerance to H_2_O_2_	[58]
FY834	Deletion of the region between *UBP2* and *LSC1* on chrXV	Sensitive to hygromycin B	[131]
FY3	Inversion of DAL cluster on chrIX	Drop in fitness in allantoin-containing medium	[132]
Strains isolated from Evolution Canyon, Israel	A rearranged 900-kb chromosome that contains two chrVIII fragments and a rearranged 650-kb chromosome containing a chrVII fragment and a chrVIII fragment.	Enhanced copper tolerance	[130]
GN	Translocation between chr XV and chrXVI involving the promoter of *ADH1* and the gene *SSU1*	Adaptation to sulfite	[134]
ScDup	Segmental duplication of region 0–158 kb on chrIII in ScDup (C3-1); 158–317 kb on chrIII in ScDup (C3-2); 398–577 kb on chrV in ScDup (C5-3); 800–1091 kb on chrVII in ScDup (C7-5); 195–403 kb on chr X in ScDup (C10-2); 491-692 kb on chr XII in ScDup (C12-3); 199–401kb on chrXV in ScDup (15-2)	Heat tolerance	[116]
ScDup	Segmental duplication of region 400–601 kb on chrII in ScDup (C2-3); 1401–1532 kb on chrIV in ScDup (C4-8)	Acetic acid tolerance	[116]
ScDup	Segmental duplication of region 0–201 kb on chrIV in ScDup (C4-1); 0–252 kb on chrXII in ScDup (C12-1); 491–692 kb on chrXII in ScDup (C12-3)	Lactic acid tolerance	[116]
ScDup	Segmental duplication of region 400–601 kb on chrII in ScDup (C2-3)	Formic acid tolerance	[116]
ScDup	Segmental duplication of region 400–601 kb on chrII in ScDup (C2-3); 0–158 kb on chrIII in ScDup (C3-1); 800–1091 kb on chrVII in ScDup (C7-5); 491–692 kb on chrXII in ScDup (C12-3); 0-205kb on chrXIII in ScDup (C13-1); 596–800 kb on chrXVI in ScDup (C16-4);	Enhanced tolerance to 1.2 M NaCl	[116]
ScDup	Segmental duplication of region 398–577 kb on chrV in ScDup (C5-3); 800–1091 kb on chrVII in ScDup (C7-5); 199–401kb on chrXV in ScDup (15-2); 198–399 kb on chrXVI in ScDup (C16-2); 596–800 kb on chrXVI in ScDup (C16-4)	Ethanol tolerance	[116]
Haploid strain with synthesized chrV	An inversion of a 7-kb region encoding *GCN4*, *YEL008W*, *MIT1,* and *YEA6* genes and deletion of a 785-bp region containing the short *MXR1* coding sequence	Improved xylose utilization	[112]
Haploid strain with synthesized chrV	Deletion of the *YEL013W-* containing region on synV	Improvement in carotenoid production	[111]
A diploid strain (crossing sake-brewing strain Y12 and a synthesized chrV-bearing strain)	Deletion of the region spanning *YJL154C*-*YJL140W*	Improved thermotolerance	[113]
Haploid strain with synthesized chrXII	An inversion involves *ZRT2* and *ACE2* on synXII	Enhanced tolerance to ethanol	[110]
A wine yeast strain	An inversion in chrXVI involves *SSU1* and *GCR1* regulatory regions	Tolerance to sulfite	[135]
Haploid strain with synthesized chrV	Deletion of the *SPT2*-containing region on synV	Enhanced alkali tolerance	[114]

## 6. Conclusions

In this review, we have discussed the origin, regulation, and evolutional effect of chromosomal rearrangements in the model organism *S. cerevisiae*. In the wild-type cells of diploid *S. cerevisiae*, the spontaneous rate of this genomic alteration is about 10^−4^ per cell division, but it can be greatly induced by DNA replication stress, DNA repair inactivation, radiation, oxidative stress, drugs, etc. Despite the various inducers, most rearrangement events in the yeast genome involve homologous recombination between dispersed repeats such as the Ty retrotransposons. It is likely that the numbers of direct repeats, the sequence similarity and distance between the repeats, and the genetic effect of the event are crucial factors to determine the patterns of chromosomal rearrangements among the various *S. cerevisiae* strains. It should be noted that the chromosomal rearmaments always occur at a much lower rate than that of LOH events under both spontaneous and stressful conditions. This phenomenon reflects that most recombinogenic lesions would be preferably repaired by SDSA and DSBR rather than other rearrangement-prone pathways in yeast (Figure 1). Moreover, most chromosomal rearrangements may be generally more harmful to cells than copy-neutral LOH events, although a specific chromosomal rearrangement can be readily selected under certain conditions. Using the technologies of introducing on-demand rearrangements, the fitness effect of a particular rearrangement event can be well explored in yeast. Additionally, the long-read sequencing has served as a powerful analysis tool to map the junctions of complex rearrangements with high resolution in yeast, as well as in higher eukaryotes. Recently, eccDNA has been shown to constitute a more prominent phenomenon of genomic instability in cancers, while the underlying genetic mechanisms of eccDNA formation remain unclear [5,6,7,141]. The excellent tools available for the yeast *S. cerevisiae* enable improved understanding of the origins and fates of eccDNA in eukaryotes.

## References

[1] Rikova K., Guo A., Zeng Q., Possemato A., Yu J., Haack H., Nardone J., Lee K., Reeves C., Li Y. (2007). Global survey of phosphotyrosine signaling identifies oncogenic kinases in lung cancer. Cell.

[2] Nebral K. (2005). NUP98 is fused to topoisomerase (DNA) II 180 kDa (TOP2B) in a patient with Acute Myeloid Leukemia with a new t(3;11)(p24;p15). Clin. Cancer Res..

[3] Paulsen T., Kumar P., Koseoglu M.M., Dutta A. (2018). Discoveries of extrachromosomal circles of DNA in normal and tumor cells. Trends Genet..

[4] Turner K.M., Deshpande V., Beyter D., Koga T., Rusert J., Lee C., Li B., Arden K., Ren B., Nathanson D.A. (2017). Extrachromosomal oncogene amplification drives tumour evolution and genetic heterogeneity. Nature.

[5] Morton A.R., Dogan-Artun N., Faber Z.J., MacLeod G., Bartels C.F., Piazza M.S., Allan K.C., Mack S.C., Wang X.X., Gimple R.C. (2019). Functional enhancers shape extrachromosomal oncogene amplifications. Cell.

[6] Wu S., Turner K.M., Nguyen N., Raviram R., Erb M., Santini J., Luebeck J., Rajkumar U., Diao Y., Li B. (2019). Circular ecDNA promotes accessible chromatin and high oncogene expression. Nature.

[7] Koche R.P., Rodriguez-Fos E., Helmsauer K., Burkert M., MacArthur I.C., Maag J., Chamorro R., Munoz-Perez N., Puiggròs M., Garcia H.D. (2020). Extrachromosomal circular DNA drives oncogenic genome remodeling in neuroblastoma. Nat. Genet..

[8] Zhang Y., Malone J.H., Powell S.K., Periwal V., Spana E., Macalpine D.M., Oliver B. (2010). Expression in aneuploid *Drosophila* S2 cells. PLoS Biol..

[9] Pologe L.G., Ravetch J.V. (1986). A chromosomal rearrangement in a *P. falciparum* histidine-rich protein gene is associated with the knobless phenotype. Nature.

[10] Selmecki A., Forche A., Berman J. (2006). Aneuploidy and isochromosome formation in drug-resistant *Candida albicans*. Science.

[11] Zhang K., Zhang L.-J., Fang Y.-H., Jin X.-N., Qi L., Wu X.-C., Zheng D.-Q. (2016). Genomic structural variation contributes to phenotypic change of industrial bioethanol yeast *Saccharomyces cerevisiae*. FEMS Yeast Res..

[12] Frankenberg-Schwager M., Frankenberg D. (1990). DNA double-strand breaks: Their repair and relationship to cell killing in yeast. Int. J. Radiat. Biol..

[13] Mimitou E.P., Symington L.S. (2008). Sae2, Exo1 and Sgs1 collaborate in DNA double-strand break processing. Nature.

[14] Zhu Z., Chung W.-H., Shim E.Y., Lee S.E., Ira G. (2008). Sgs1 helicase and two nucleases Dna2 and Exo1 resect DNA double-strand break ends. Cell.

[15] Zakharyevich K., Ma Y., Tang S., Hwang P.Y.-H., Boiteux S., Hunter N. (2010). Temporally and biochemically distinct activities of Exo1 during meiosis: Double-strand break resection and resolution of double holliday junctions. Mol. Cell.

[16] Lichten M., Chung W.-H., Zhu Z., Papusha A., Malkova A., Ira G. (2010). Defective resection at DNA double-strand breaks leads to de novo telomere formation and enhances gene targeting. PLoS Genet..

[17] Gravel S., Chapman J.R., Magill C., Jackson S.P. (2008). DNA helicases Sgs1 and BLM promote DNA double-strand break resection. Genes Dev..

[18] Deng S.K., Yin Y., Petes T.D., Symington L.S. (2015). Mre11-Sae2 and RPA Collaborate to Prevent Palindromic Gene Amplification. Mol. Cell.

[19] Piazza A., Wright W.D., Heyer W.D. (2017). Multi-invasions Are Recombination Byproducts that Induce Chromosomal Rearrangements. Cell.

[20] Chang H.H.Y., Pannunzio N.R., Adachi N., Lieber M.R. (2017). Non-homologous DNA end joining and alternative pathways to double-strand break repair. Nat. Rev. Mol. Cell Biol..

[21] Mimitou E.P., Symington L.S. (2010). Ku prevents Exo1 and Sgs1-dependent resection of DNA ends in the absence of a functional MRX complex or Sae2. EMBO J..

[22] Pannunzio N.R., Watanabe G., Lieber M.R. (2018). Nonhomologous DNA end-joining for repair of DNA double-strand breaks. J. Biol. Chem..

[23] Meyer D., Fu B.X.H., Heyer W.-D. (2015). DNA polymerases δ and λ cooperate in repairing double-strand breaks by microhomology-mediated end-joining in *Saccharomyces cerevisiae*. Proc. Natl. Acad. Sci. USA.

[24] McVey M., Lee S.E. (2008). MMEJ repair of double-strand breaks (director’s cut): Deleted sequences and alternative endings. Trends Genet..

[25] Bell J.C., Kowalczykowski S.C. (2016). RecA: Regulation and Mechanism of a Molecular Search Engine. Trends Biochem. Sci..

[26] Scully R., Panday A., Elango R., Willis N.A. (2019). DNA double-strand break repair-pathway choice in somatic mammalian cells. Nat. Rev. Mol. Cell Biol..

[27] Jachimowicz R.D., Goergens J., Reinhardt H.C. (2019). DNA double-strand break repair pathway choice—from basic biology to clinical exploitation. Cell Cycle.

[28] Ceccaldi R., Rondinelli B., D’Andrea A.D. (2016). Repair pathway choices and consequences at the double-strand break. Trends Cell Biol..

[29] Han J., Huang J. (2020). DNA double-strand break repair pathway choice: The fork in the road. Genome Instab. Dis..

[30] Xu Y., Xu D. (2020). Repair pathway choice for double-strand breaks. Essays Biochem..

[31] Lehner K., Mudrak S.V., Minesinger B.K., Jinks-Robertson S. (2012). Frameshift mutagenesis: The roles of primer-template misalignment and the nonhomologous end-joining pathway in *Saccharomyces cerevisiae*. Genetics.

[32] Cho J.-E., Jinks-Robertson S. (2019). Deletions associated with stabilization of the Top1 cleavage complex in yeast are products of the nonhomologous end-joining pathway. Proc. Natl. Acad. Sci. USA.

[33] Bhargava R., Carson C.R., Lee G., Stark J.M. (2017). Contribution of canonical nonhomologous end joining to chromosomal rearrangements is enhanced by ATM kinase deficiency. Proc. Natl. Acad. Sci. USA.

[34] Sunder S., Wilson T.E. (2019). Frequency of DNA end joining in trans is not determined by the predamage spatial proximity of double-strand breaks in yeast. Proc. Natl. Acad. Sci. USA.

[35] Ahrabi S., Sarkar S., Pfister S.X., Pirovano G., Higgins G.S., Porter A.C., Humphrey T.C. (2016). A role for human homologous recombination factors in suppressing microhomology-mediated end joining. Nucleic Acids Res..

[36] Wyatt D.W., Feng W., Conlin M.P., Yousefzadeh M.J., Roberts S.A., Mieczkowski P., Wood R.D., Gupta G.P., Ramsden D.A. (2016). Essential Roles for Polymerase θ-Mediated End Joining in the Repair of Chromosome Breaks. Mol. Cell.

[37] Symington L.S., Sinha S., Li F., Villarreal D., Shim J.H., Yoon S., Myung K., Shim E.Y., Lee S.E. (2017). Microhomology-mediated end joining induces hypermutagenesis at breakpoint junctions. PLoS Genet..

[38] Vaze M.B., Pellicioli A., Lee S.E., Ira G., Haber J.E. (2002). Recovery from checkpoint-mediated arrest after repair of a double-strand break requires Srs2 helicase. Mol. Cell.

[39] Kirkpatrick D.T., Manthey G.M., Bailis A.M. (2010). Rad51 Inhibits Translocation Formation by Non-Conservative Homologous Recombination in *Saccharomyces cerevisiae*. PLoS ONE.

[40] Argueso J.L., Westmoreland J., Mieczkowski P.A., Gawel M., Petes T.D., Resnick M.A. (2008). Double-strand breaks associated with repetitive DNA can reshape the genome. Proc. Natl. Acad. Sci. USA.

[41] Ramakrishnan S., Kockler Z., Evans R., Downing B.D., Malkova A. (2018). Single-strand annealing between inverted DNA repeats: Pathway choice, participating proteins, and genome destabilizing consequences. PLoS Genet..

[42] Li B.Z., Putnam C.D., Kolodner R.D. (2020). Mechanisms underlying genome instability mediated by formation of foldback inversions in *Saccharomyces cerevisiae*. Elife.

[43] Symington L.S., Rothstein R., Lisby M. (2014). Mechanisms and regulation of mitotic recombination in *Saccharomyces cerevisiae*. Genetics.

[44] Sansregret L., Swanton C. (2017). The role of aneuploidy in cancer evolution. Cold Spring Harb. Persp. Med..

[45] Piazza A., Heyer W.D. (2019). Homologous Recombination and the Formation of Complex Genomic Rearrangements. Trends Cell Biol..

[46] Hastings P.J., Lupski J.R., Rosenberg S.M., Ira G. (2009). Mechanisms of change in gene copy number. Nat. Rev. Genet..

[47] Kramara J., Osia B., Malkova A. (2018). Break-induced replication: The where, the why, and the how. Trends Genet..

[48] Hastings P.J., Vasan S., Deem A., Ramakrishnan S., Argueso J.L., Malkova A. (2014). Cascades of Genetic Instability Resulting from Compromised Break-Induced Replication. PLoS Genet..

[49] Piazza A., Heyer W.-D. (2018). Multi-Invasion-Induced Rearrangements as a Pathway for Physiological and Pathological Recombination. Bioessays.

[50] Heyer W.-D. (2015). Regulation of Recombination and Genomic Maintenance. Cold Spring Harb. Perspect. Biol..

[51] Myung K., Kolodner R.D. (2003). Induction of genome instability by DNA damage in *Saccharomyces cerevisiae*. DNA Repair.

[52] Fasullo M., Dave P., Rothstein R. (1994). DNA-damaging agents stimulate the formation of directed reciprocal translocations in *Saccharomyces cerevisiae*. Mutat. Res..

[53] Sui Y., Qi L., Wu J.K., Wen X.P., Tang X.X., Ma Z.J., Wu X.C., Zhang K., Kokoska R.J., Zheng D.Q. (2020). Genome-wide mapping of spontaneous genetic alterations in diploid yeast cells. Proc. Natl. Acad. Sci. USA.

[54] Zhu Y.O., Siegal M.L., Hall D.W., Petrov D.A. (2014). Precise estimates of mutation rate and spectrum in yeast. Proc. Natl. Acad. Sci. USA.

[55] Sampaio N.M.V., Ajith V., Watson R.A., Heasley L.R., Chakraborty P., Rodrigues-Prause A., Malc E.P., Mieczkowski P.A., Nishant K.T., Argueso J.L. (2020). Characterization of systemic genomic instability in budding yeast. Proc. Natl. Acad. Sci. USA.

[56] Loeillet S., Herzog M., Puddu F., Legoix P., Baulande S., Jackson S.P., Nicolas A.G. (2020). Trajectory and uniqueness of mutational signatures in yeast mutators. Proc. Natl. Acad. Sci. USA.

[57] St Charles J., Petes T.D. (2013). High-resolution mapping of spontaneous mitotic recombination hotspots on the 1.1 Mb arm of yeast chromosome IV. PLoS Genet..

[58] Zhang K., Zheng D.Q., Sui Y., Qi L., Petes T.D. (2019). Genome-wide analysis of genomic alterations induced by oxidative DNA damage in yeast. Nucleic Acids Res..

[59] Zhou Z.-X., Lujan S.A., Burkholder A.B., Garbacz M.A., Kunkel T.A. (2019). Roles for DNA polymerase δ in initiating and terminating leading strand DNA replication. Nat. Commun..

[60] Garbacz M.A., Cox P.B., Sharma S., Lujan S.A., Chabes A., Kunkel T.A. (2019). The absence of the catalytic domains of *Saccharomyces cerevisiae* DNA polymerase ϵ strongly reduces DNA replication fidelity. Nucleic Acids Res..

[61] Zheng D.-Q., Petes T.D. (2018). Genome instability induced by low levels of replicative DNA polymerases in yeast. Genes.

[62] Ubhi T., Brown G.W. (2019). Exploiting DNA replication stress for cancer treatment. Cancer Res..

[63] Maffia A., Ranise C., Sabbioneda S. (2020). From R-Loops to G-Quadruplexes: Emerging New Threats for the Replication Fork. Int. J. Mol. Sci..

[64] Sui Y., Qi L., Zhang K., Saini N., Klimczak L.J., Sakofsky C.J., Gordenin D.A., Petes T.D., Zheng D.-Q. (2020). Analysis of APOBEC-induced mutations in yeast strains with low levels of replicative DNA polymerases. Proc. Natl. Acad. Sci. USA.

[65] Zheng D.Q., Zhang K., Wu X.C., Mieczkowski P.A., Petes T.D. (2016). Global analysis of genomic instability caused by DNA replication stress in *Saccharomyces cerevisiae*. Proc. Natl. Acad. Sci. USA.

[66] Lemoine F.J., Degtyareva N.P., Lobachev K., Petes T.D. (2005). Chromosomal translocations in yeast induced by low levels of DNA polymerase: A model for chromosome fragile sites. Cell.

[67] Kokoska R.J., Stefanovic L., DeMai J., Petes T.D. (2000). Increased rates of genomic deletions generated by mutations in the yeast gene encoding DNA polymerase delta or by decreases in the cellular levels of DNA polymerase delta. Mol. Cell. Biol..

[68] Salim D., Bradford W.D., Freeland A., Cady G., Wang J., Pruitt S.C., Gerton J.L. (2017). DNA replication stress restricts ribosomal DNA copy number. PLoS Genet..

[69] Srivatsan A., Li B., Sanchez D.N., Somach S.B., da Silva V.L., de Souza S.J., Putnam C.D., Kolodner R.D. (2019). Essential *Saccharomyces cerevisiae* genome instability suppressing genes identify potential human tumor suppressors. Proc. Natl. Acad. Sci. USA.

[70] Bantele S.C.S., Lisby M., Pfander B. (2019). Quantitative sensing and signalling of single-stranded DNA during the DNA damage response. Nat. Commun..

[71] Deshpande I., Seeber A., Shimada K., Keusch J.J., Gut H., Gasser S.M. (2017). Structural Basis of Mec1-Ddc2-RPA Assembly and Activation on Single-Stranded DNA at Sites of Damage. Mol. Cell.

[72] Menin L., Colombo C.V., Maestrini G., Longhese M.P., Clerici M. (2019). Tel1/ATM Signaling to the Checkpoint Contributes to Replicative Senescence in the Absence of Telomerase. Genetics.

[73] McCulley J.L., Petes T.D. (2010). Chromosome rearrangements and aneuploidy in yeast strains lacking both Tel1p and Mec1p reflect deficiencies in two different mechanisms. Proc. Natl. Acad. Sci. USA.

[74] Myung K., Datta A., Kolodner R.D.J.C. (2001). Suppression of Spontaneous Chromosomal Rearrangements by S Phase Checkpoint Functions in *Saccharomyces cerevisiae*. Cell.

[75] Putnam C.D., Pallis K., Hayes T.K., Kolodner R.D. (2014). DNA repair pathway selection caused by defects in TEL1, SAE2, and de novo telomere addition generates specific chromosomal rearrangement signatures. PLoS Genet..

[76] Putnam C.D., Kolodner R.D. (2017). Pathways and Mechanisms that Prevent Genome Instability in *Saccharomyces cerevisiae*. Genetics.

[77] Putnam C.D., Hayes T.K., Kolodner R.D. (2009). Specific pathways prevent duplication-mediated genome rearrangements. Nature.

[78] Chen C., Kolodner R.D. (1999). Gross chromosomal rearrangements in *Saccharomyces cerevisiae* replication and recombination defective mutants. Nat. Genet..

[79] Myung K., Datta A., Chen C., Kolodner R.D. (2001). SGS1, the *Saccharomyces cerevisiae* homologue of BLM and WRN, suppresses genome instability and homeologous recombination. Nat. Genet..

[80] Doerfler L., Schmidt K.H. (2014). Exo1 phosphorylation status controls the hydroxyurea sensitivity of cells lacking the Pol32 subunit of DNA polymerases delta and zeta. DNA Repair.

[81] Campos-Doerfler L., Syed S., Schmidt K.H. (2018). Sgs1 Binding to Rad51 Stimulates Homology-Directed DNA Repair in *Saccharomyces cerevisiae*. Genetics.

[82] Kaniecki K., De Tullio L., Gibb B., Kwon Y., Sung P., Greene E.C. (2017). Dissociation of Rad51 Presynaptic Complexes and Heteroduplex DNA Joints by Tandem Assemblies of Srs2. Cell Rep..

[83] Fabre F., Chan A., Heyer W.D., Gangloff S. (2002). Alternate pathways involving Sgs1/Top3, Mus81/ Mms4, and Srs2 prevent formation of toxic recombination intermediates from single-stranded gaps created by DNA replication. Proc. Natl. Acad. Sci. USA.

[84] Prakash R., Satory D., Dray E., Papusha A., Scheller J., Kramer W., Krejci L., Klein H., Haber J.E., Sung P. (2009). Yeast Mph1 helicase dissociates Rad51-made D-loops: Implications for crossover control in mitotic recombination. Genes Dev..

[85] Fasching C.L., Cejka P., Kowalczykowski S.C., Heyer W.D. (2015). Top3-Rmi1 dissolve Rad51-mediated D loops by a topoisomerase-based mechanism. Mol. Cell.

[86] Chakraborty U., Alani E. (2016). Understanding how mismatch repair proteins participate in the repair/anti-recombination decision. FEMS Yeast Res..

[87] Hum Y.F., Jinks-Robertson S. (2019). Mismatch recognition and subsequent processing have distinct effects on mitotic recombination intermediates and outcomes in yeast. Nucleic Acids Res..

[88] Muraki K., Han L., Miller D., Murnane J.P. (2015). Processing by MRE11 is involved in the sensitivity of subtelomeric regions to DNA double-strand breaks. Nucleic Acids Res..

[89] Kupiec M. (2000). Damage-induced recombination in the yeast *Saccharomyces cerevisiae*. Mutat. Res..

[90] Boiteux S., Jinks-Robertson S. (2013). DNA repair mechanisms and the bypass of DNA damage in *Saccharomyces cerevisiae*. Genetics.

[91] Arbel M., Liefshitz B., Kupiec M. (2020). DNA damage bypass pathways and their effect on mutagenesis in yeast. FEMS Microbiol. Rev..

[92] Yin Y., Petes T.D. (2013). Genome-wide high-resolution mapping of UV-induced mitotic recombination events in *Saccharomyces cerevisiae*. PLoS Genet..

[93] Hoffmann G.R., Laterza A.M., Sylvia K.E., Tartaglione J.P. (2011). Potentiation of the mutagenicity and recombinagenicity of bleomycin in yeast by unconventional intercalating agents. Environ. Mol. Mutagen..

[94] Freeman K.M., Hoffmann G.R. (2007). Frequencies of mutagen-induced coincident mitotic recombination at unlinked loci in *Saccharomyces cerevisiae*. Mutat. Res. Fundam. Mol. Mech. Mutagen..

[95] Sheng H., Qi L., Sui Y., Li Y.Z., Yu L.Z., Zhang K., Xu J.Z., Wang P.M., Zheng D.Q. (2019). Mapping chromosomal instability induced by small-molecular therapeutics in a yeast model. Appl. Microbiol. Biotechnol..

[96] Gittens W.H., Johnson D.J., Allison R.M., Cooper T.J., Thomas H., Neale M.J. (2019). A nucleotide resolution map of Top2-linked DNA breaks in the yeast and human genome. Nat. Commun..

[97] Tombline G., Millen J., Polevoda B., Rapaport M., Baxter B., Van Meter M., Gilbertson M., Madrey J., Piazza G.A., Rasmussen L.J.A. (2017). Effects of an unusual poison identify a lifespan role for Topoisomerase 2 in *Saccharomyces cerevisiae*. Aging.

[98] Morimoto S., Tsuda M., Bunch H., Sasanuma H., Austin C., Takeda S. (2019). Type II DNA Topoisomerases Cause Spontaneous Double-Strand Breaks in Genomic DNA. Genes.

[99] Sloan R., Huang S.-Y.N., Pommier Y., Jinks-Robertson S. (2017). Effects of camptothecin or TOP1 overexpression on genetic stability in *Saccharomyces cerevisiae*. DNA Repair.

[100] Andersen S.L., Sloan R.S., Petes T.D., Jinks-Robertson S. (2015). Genome-destabilizing effects associated with top1 loss or accumulation of top1 cleavage complexes in yeast. PLoS Genet..

[101] Shen L., Wang Y.-T., Tang X.-X., Zhang K., Wang P.-M., Sui Y., Zheng D.-Q. (2020). Heat shock drives genomic instability and phenotypic variations in yeast. AMB Express.

[102] Zheng D.Q., Jin X.N., Zhang K., Fang Y.H., Wu X.C. (2017). Novel strategy to improve vanillin tolerance and ethanol fermentation performances of *Saccharomycere cerevisiae* strains. Bioresour. Technol..

[103] Zhang K., Tong M., Gao K., Di Y., Wang P., Zhang C., Wu X., Zheng D. (2015). Genomic reconstruction to improve bioethanol and ergosterol production of industrial yeast *Saccharomyces cerevisiae*. J. Ind. Microbiol. Biotechnol..

[104] Zheng D.-Q., Chen J., Zhang K., Gao K.-H., Li O., Wang P.-M., Zhang X.-Y., Du F.-G., Sun P.-Y., Qu A.-M. (2013). Genomic structural variations contribute to trait improvement during whole-genome shuffling of yeast. Appl. Microbiol. Biotechnol..

[105] Qi L., Wu X.C., Zheng D.Q. (2019). Hydrogen peroxide, a potent inducer of global genomic instability. Curr. Genet..

[106] Cadet J., Davies K.J. (2017). Oxidative DNA damage & repair: An introduction. Free Radic. Biol. Med..

[107] Huang M.-E., Kolodner R.D. (2005). A biological network in *Saccharomyces cerevisiae* prevents the deleterious effects of endogenous oxidative DNA damage. Mol. Cell.

[108] Qi L., Zhang K., Wang Y.T., Wu J.K., Sui Y., Liang X.Z., Yu L.Z., Wu X.C., Wang P.M., Xu J.Z. (2019). Global Analysis of Furfural-Induced Genomic Instability Using a Yeast Model. Appl. Environ. Microbiol..

[109] Richardson S.M., Mitchell L.A., Stracquadanio G., Yang K., Dymond J.S., DiCarlo J.E., Lee D., Huang C.L.V., Chandrasegaran S., Cai Y. (2017). Design of a synthetic yeast genome. Science.

[110] Luo Z., Wang L., Wang Y., Zhang W., Guo Y., Shen Y., Jiang L., Wu Q., Zhang C., Cai Y. (2018). Identifying and characterizing SCRaMbLEd synthetic yeast using ReSCuES. Nat. Commun..

[111] Jia B., Wu Y., Li B.Z., Mitchell L.A., Liu H., Pan S., Wang J., Zhang H.R., Jia N., Li B. (2018). Precise control of SCRaMbLE in synthetic haploid and diploid yeast. Nat. Commun..

[112] Blount B.A., Gowers G.F., Ho J.C.H., Ledesma-Amaro R., Jovicevic D., McKiernan R.M., Xie Z.X., Li B.Z., Yuan Y.J., Ellis T. (2018). Rapid host strain improvement by in vivo rearrangement of a synthetic yeast chromosome. Nat. Commun..

[113] Shen M.J., Wu Y., Yang K., Li Y., Xu H., Zhang H., Li B.Z., Li X., Xiao W.H., Zhou X. (2018). Heterozygous diploid and interspecies SCRaMbLEing. Nat. Commun..

[114] Ma L., Li Y., Chen X., Ding M., Wu Y., Yuan Y.J. (2019). SCRaMbLE generates evolved yeasts with increased alkali tolerance. Microb. Cell Fact..

[115] Fleiss A., O’Donnell S., Fournier T., Lu W., Agier N., Delmas S., Schacherer J., Fischer G. (2019). Reshuffling yeast chromosomes with CRISPR/Cas9. PLoS Genet..

[116] Natesuntorn W., Iwami K., Matsubara Y., Sasano Y., Sugiyama M., Kaneko Y., Harashima S. (2015). Genome-wide construction of a series of designed segmental aneuploids in *Saccharomyces cerevisiae*. Sci. Rep..

[117] Charles J.S., Hazkani-Covo E., Yin Y., Andersen S.L., Dietrich F.S., Greenwell P.W., Malc E., Mieczkowski P., Petes T.D. (2012). High-resolution Genome-wide Analysis of Irradiated (UV and gamma rays) Diploid Yeast Cells Reveals a High Frequency of Genomic Loss of Heterozygosity (LOH) Events. Genetics.

[118] McGinty R.J., Rubinstein R.G., Neil A.J., Dominska M., Kiktev D., Petes T.D., Mirkin S.M. (2017). Nanopore sequencing of complex genomic rearrangements in yeast reveals mechanisms of repeat-mediated double-strand break repair. Genome Res..

[119] Payne A., Holmes N., Rakyan V., Loose M., Birol I. (2019). BulkVis: A graphical viewer for Oxford nanopore bulk FAST5 files. Bioinformatics.

[120] Wick R.R., Judd L.M., Holt K.E. (2019). Performance of neural network basecalling tools for Oxford Nanopore sequencing. Genome Biol..

[121] Sedlazeck F.J., Rescheneder P., Smolka M., Fang H., Nattestad M., Von Haeseler A., Schatz M.C.J.N.M. (2018). Accurate detection of complex structural variations using single-molecule sequencing. Nat. Methods.

[122] Nattestad M., Aboukhalil R., Chin C.-S., Schatz M.C. (2020). Ribbon: Intuitive visualization for complex genomic variation. Bioinformatics.

[123] Ho S.S., Urban A.E., Mills R.E. (2020). Structural variation in the sequencing era. Nat. Rev. Genet..

[124] Qin L., Dong S., Yu J., Ning X., Xu K., Zhang S.J., Xu L., Li B.Z., Li J., Yuan Y.J. (2020). Stress-driven dynamic regulation of multiple tolerance genes improves robustness and productive capacity of *Saccharomyces cerevisiae* in industrial lignocellulose fermentation. Metab. Eng..

[125] Auesukaree C. (2017). Molecular mechanisms of the yeast adaptive response and tolerance to stresses encountered during ethanol fermentation. J. Biosci. Bioeng..

[126] Gilchrist C., Stelkens R. (2019). Aneuploidy in yeast: Segregation error or adaptation mechanism?. Yeast.

[127] Jin J., Jia B., Yuan Y.-J. (2020). Yeast chromosomal engineering to improve industrially-relevant phenotypes. Curr. Opin. Biotechnol..

[128] Hose J., Escalante L.E., Clowers K.J., Dutcher H.A., Robinson D., Bouriakov V., Coon J.J., Shishkova E., Gasch A.P. (2020). The genetic basis of aneuploidy tolerance in wild yeast. Elife.

[129] Sun X., Liu L., Zhao Y., Ma T., Zhao F., Huang W., Zhan J. (2016). Effect of copper stress on growth characteristics and fermentation properties of *Saccharomyces cerevisiae* and the pathway of copper adsorption during wine fermentation. Food Chem..

[130] Chang S.L., Lai H.Y., Tung S.Y., Leu J.Y. (2013). Dynamic large-scale chromosomal rearrangements fuel rapid adaptation in yeast populations. PLoS Genet..

[131] Sugiyama M., Nakazawa T., Murakami K., Sumiya T., Nakamura A., Kaneko Y., Nishizawa M., Harashima S. (2008). PCR-mediated one-step deletion of targeted chromosomal regions in haploid *Saccharomyces cerevisiae*. Appl. Microbiol. Biotechnol..

[132] Naseeb S., Delneri D. (2012). Impact of chromosomal inversions on the yeast DAL cluster. PLoS ONE.

[133] Nadai C., Treu L., Campanaro S., Giacomini A., Corich V. (2016). Different mechanisms of resistance modulate sulfite tolerance in wine yeasts. Appl. Microbiol. Biotechnol..

[134] Zimmer A., Durand C., Loira N., Durrens P., Sherman D.J., Marullo P. (2014). QTL dissection of Lag phase in wine fermentation reveals a new translocation responsible for *Saccharomyces cerevisiae* adaptation to sulfite. PLoS ONE.

[135] García-Ríos E., Nuévalos M., Barrio E., Puig S., Guillamón J.M. (2019). A new chromosomal rearrangement improves the adaptation of wine yeasts to sulfite. Environ. Microbiol..

[136] Dunham M.J., Badrane H., Ferea T., Adams J., Brown P.O., Rosenzweig F., Botstein D. (2002). Characteristic genome rearrangements in experimental evolution of *Saccharomyces cerevisiae*. Proc. Natl. Acad. Sci. USA.

[137] Gresham D., Desai M.M., Tucker C.M., Jenq H.T., Pai D.A., Ward A., DeSevo C.G., Botstein D., Dunham M.J. (2008). The repertoire and dynamics of evolutionary adaptations to controlled nutrient-limited environments in yeast. PLoS Genet..

[138] Hunt C.R., Sim J.E., Sullivan S.J., Featherstone T., Golden W., Von Kapp-Herr C., Hock R.A., Gomez R.A., Parsian A.J., Spitz D.R. (1998). Genomic instability and catalase gene amplification induced by chronic exposure to oxidative stress. Cancer Res..

[139] Zhu J., Tsai H.J., Gordon M.R., Li R. (2018). Cellular Stress Associated with Aneuploidy. Dev. Cell.

[140] Zhou L., Jilderda L.J., Foijer F. (2020). Exploiting aneuploidy-imposed stresses and coping mechanisms to battle cancer. Open Biol..

[141] Hull R.M., King M., Pizza G., Krueger F., Vergara X., Houseley J. (2019). Transcription-induced formation of extrachromosomal DNA during yeast ageing. PLoS Biol..

